# Notes and News

**Published:** 1901-05-11

**Authors:** 


### Notes and News.

A hospital for the reception of patients suffering from incurable cancer, and an investigation institute is to be established in Moscow.

Dr. Robert Hutchinson is going to give a course of lectures on "Foods and Dietetics" on Wednesdays and Fridays, commencing May 8th, at the London Hospital Medical College.

A Cottage Hospital has been opened at Carnarvon. The wards are situated in two wings, communicating by corridors with the Central Administration block. Each ward contains six beds. Mr. Rowland Lloyd Jones, of Carnarvon, is the architect.

New medical legislation for Belgium is under consideration. The proposals embrace regulations for the training and practice of midwives, which are very definite. The examiners for the diplomas are to be appointed by Government, which also defines the curriculum.

The authorities in India are again having trouble with the natives in respect to cholera precautions. In the Sialkote district, near Cashmere, a native subaltern has been killed and several hospital assistants have been attacked. It has been found necessary to send further military aid.

At a meeting of the special election committee of the Liverpool Royal Infirmary, held on the 2nd instant, they elected as one of the assistant honorary physicians to the institution, Dr. Robert J. M. Buchanan, M.D., B.Ch.Vict., M.R.C.P.Lond., &c., at present physician to the Stanley, and assistant physician to the consumption hospitals, and assistant lecturer on Forensic Medicine at University College, Liverpool. There were six other candidates, all also very highly qualified, but Dr. Buchanan was the senior one, and had greater claims on the electors, having formerly held the posts of house physician, house surgeon, gynæcological house surgeon, medical tutor, and surgical tutor at the hospital, besides demonstratorships of pathology and physiology at university colleges with which the hospital is associated for teaching purposes.

Princess Henry of Battenberg has presented a memorial to be erected to the late Major G. Hilliard, R.A.M.C., in South Africa. The inscription includes the words: "Erected in grateful remembrance of the care he rendered her husband, by Beatrice, Princess Henry of Battenberg."

The Cremation Bill is now before Parliament. It is for the purpose of regulating cremation, and to enable the burial authorities to establish crematoria. One clause was amended and put in the Bill that no crematorium should be used until certified by the burial authority to the Home Secretary that it is complete and properly equipped. Another amendment provides for the registration of cremations.

The foundation stone of a new sanatorium has been laid near Greenhill for the county of Dorset, by the Duchess of Beaufort. The new institution owes its existence to the energy of Dr. Macpherson Lawrie, medical superintendent of the original sanatorium. The building will contain four wards and two small special wards, day rooms, out-patients' department, and the usual offices. The estimate is for £14,000.

The retirement of Sir Edwin Galsworthy from the chairmanship of the Metropolitan Asylums Board being now definitely decided upon, at the last meeting of the Board a resolution was passed expressing the regret of the members in losing their valued colleague, and testifying to the able manner in which he had carried out the duties of his office. Although Sir Edwin is obliged for health reasons to relinquish the arduous post of chairman, he does not intend to sever his connection with the Board.

We are officially informed that the Committee of Inquiry into the dispute between the Medical Staff and the Board of Management, the position, functions, and acts of the Secretary-Director, and the constitution, rules, and management of the National Hospital for the Paralysed and Epileptic, consists of the following gentlemen:—Sir Edward Fry, chairman; Lord Wolverton; Sir William Karslake, K.C.; Mr. Timothy Holmes, and Mr. Cecil Henry Russell. Mr. J. Danvers Power will act as hon. secretary to the committee.

There is no walk in life which does not become, sooner or later, an object for the benevolent to establish a "Home." The latest scheme comes from America, where a medical man is endeavouring to establish a "Home" for destitute and superannuated members of the medical profession. There can be only one advantage in these "class" communities, and that is the facilities of appeal which such a scheme affords. The benevolent interested in the medical profession will no doubt come forward with their money, but is it kind to herd together 250 medical men (which is the number the scheme we refer to provides for) away from their friends? The cost of establishment alone would produce £16 per annum for each proposed beneficiary. Add this sum to the necessary cost of maintenance, and a nice little pension would be produced which might be spent without interference with the liberty of the subject, and would be in our estimation a far more welcome way of helping impoverished medical men than the offer of any community residence, however comfortable.

